# Preliminary Survey Insights on Neurologists’ Views of a Potential Oral, Safe Disease‐Modifying Therapy for Early Alzheimer’s Disease

**DOI:** 10.1002/alz70861_108480

**Published:** 2025-12-23

**Authors:** Tianyang Xi, Fred Kim, Vitella Fomenko, David R Greeley, Jai Jun Choung

**Affiliations:** ^1^ AriBio Co., Ltd., San Diego, CA USA; ^2^ AriBio Co., Ltd., Seongnam‐si, Gyeonggi‐do Korea, Republic of (South)

## Abstract

**Background:**

Alzheimer’s disease (AD) already affects >55 million people and is projected to triple by 2050, posing substantial clinical and socioeconomic burdens. Although anti‐amyloid monoclonal antibodies modestly delay cognitive decline, their intravenous delivery, intensive monitoring, and amyloid‐related imaging abnormality (ARIA) risk limit broad use. Clinicians are therefore seeking disease‐modifying treatments (DMTs) that couple robust efficacy with greater safety and ease of administration. An oral small‐molecule DMT could meet this need by improving adherence and access while providing meaningful clinical benefit. We surveyed neurologists to characterize remaining therapeutic gaps and gauge receptivity to such a therapy for early‐stage AD.

**Method:**

An online, cross‐sectional survey was conducted from 7–16 July 2024 among prescribing neurologists in the United States and Europe. Eligibility required active practice, ≥20 AD patients/month (≥20 % mild cognitive impairment or mild AD), familiarity with approved AD drugs, and documented AD expertise. Respondents reviewed an anonymized target profile describing a once‐daily oral small‐molecule with multimodal mechanisms, favorable Phase 2 safety, and cognitive plus plasma pTau181 signals. The questionnaire captured perceptions of current treatments and projected prescribing and patient acceptance rates for the potential therapy.

**Result:**

Thirty neurologists completed the survey (US n = 18; EU n = 12). First‐line symptomatic agents (cholinesterase inhibitors, memantine) were judged to provide modest benefit, with side effect–limited compliance of 60–90 %. Recently approved antibodies were further constrained by ARIA risk and infusion logistics. Twenty‐seven respondents (90 %) indicated they would prescribe the oral DMT, planning to offer it to 57 % of eligible patients; projected acceptance averaged 76 %. Top prescribing drivers were anticipated disease‐modifying effect, oral dosing convenience, safety profile, and cost.

**Conclusion:**

This study delivers a quantitative benchmark of clinician‐defined uptake thresholds for an oral small‐molecule DMT in early AD. Neurologists’ willingness to treat more than half of eligible patients—substantially exceeding current infusion uptake—highlights the potential systemwide value of a safe, orally delivered therapy. These clinician‐derived targets can inform Phase 3 trial design, payer value models, and implementation strategies, accelerating the translation of lower‐burden DMTs that may ease caregiver load and deliver renewed hope to people living with AD and their families.

